# Towards a re-emergence of chloroquine sensitivity in Côte d’Ivoire?

**DOI:** 10.1186/s12936-018-2551-7

**Published:** 2018-11-07

**Authors:** Oléfongo Dagnogo, Aristide Berenger Ako, Lacinan Ouattara, Noel Dougba Dago, David N’golo Coulibaly, André Offianan Touré, Joseph Allico Djaman

**Affiliations:** 1 UFR Biosciences, Félix Houphouët-Boigny University, BP V 34, Abidjan 01, Côte d’Ivoire; 2 Institut Pasteur of Côte d’Ivoire, 01 BP 490, Abidjan 01, Côte d’Ivoire; 3 Department of Food Science and Technology, Nangui Abrogoua University, 02 BP 801, Abidjan 02, Côte d’Ivoire; 4 UFR Sciences Biologiques, Péléforo Gon Coulibaly University, BP1328 Korhogo, Côte d’Ivoire

**Keywords:** *Pfcrt*, Thr-76, Chloroquine sensitivity, Côte d’Ivoire, Antimalarial drug resistance

## Abstract

**Background:**

Resistance of *Plasmodium falciparum* to anti-malarial drugs has hampered efforts to eradicate malaria. Recent reports of a decline in the prevalence of chloroquine-resistant *P. falciparum* in several countries, including Malawi and Zambia, is raising the hope of reintroducing chloroquine in the near future, ideally in combination with another anti-malarial drug for the treatment of uncomplicated malaria. In Côte d’Ivoire, the decrease in the clinical efficacy of chloroquine, in addition to a high proportion of clinical isolates carrying the Thr-76 mutant allele of the *pfcrt* gene, had led to the discontinuation of the use of chloroquine in 2004. Previous studies have indicated the persistence of a high prevalence of the Thr-76 mutant allele despite the withdrawal of chloroquine as first-line anti-malarial drug. This present study is conducted to determine the prevalence of the Thr-76T mutant allele of the Pfcrt gene after a decade of the ban on the sale and use of chloroquine in Côte d’Ivoire.

**Results:**

Analysis of the 64 sequences from all three study sites indicated a prevalence of 15% (10/64) of the Thr-76 mutant allele against 62% (40/64) of the Lys-76 wild-type allele. No mutation of the allele Thr-76 was observed at Anonkoua Kouté while this mutant allele was in 31% (5/16) and 25% (5/20) of isolate sequences from Port-Bouët and Ayamé respectively.

**Conclusion:**

More than a decade after the discontinuation of the use of chloroquine in Côte d’Ivoire, the proportion of parasites sensitive to this anti-malarial seems to increase in Anonkoua-kouté, Port-bouët and Ayamé.

**Electronic supplementary material:**

The online version of this article (10.1186/s12936-018-2551-7) contains supplementary material, which is available to authorized users.

## Background

Malaria remains a major public health problem in the world. According to the World Health Organization (WHO), 212 million cases of malaria were recorded in 2015, of which 429,000 led to death, of which 92% occurred in Africa with 70% of children under 5 years of age [1]. Up to 1990, chloroquine (CQ) was the main malaria treatment therapy thanks to its efficacy, safety, low cost and antipyretic properties. In the late 1950s, resistance to CQ emerged in different parts of the world, first in South-East Asia and South America (Colombia and Venezuela) [2–5]. The resistance to CQ spread rapidly and was detected in West Africa in the 80s and 90s [6, 7].

In Côte d’Ivoire, the prevalence of CQ-resistant parasites that used to be very low in 1987 with only three confirmed cases of chloroquine resistance (CQR) appeared to have increased [8, 9]. Indeed, high prevalence of the K76T mutation, a key mutation associated with *Plasmodium falciparum* resistance to CQ, have been reported in Yopougon in Abidjan (65%), Bonoua (100%), Samo (95%) and Adzopé (62%) [10]. Recent reports of a decline in the prevalence of chloroquine-resistant *P. falciparum* in several countries, including Malawi and Zambia, raise the hope of reintroducing chloroquine in the near future ideally in combination with another anti-malarial drug for the treatment of uncomplicated malaria [11, 12]. It could possibly be given to non-vulnerable groups, but it requires close monitoring of possible reemergence of CQ resistance development. Indeed, it has been suggested that effective and sustained withdrawal of CQ could lead to the reappearance of CQ-sensitive *P. falciparum*. It is the case in East Africa, particularly in Malawi and Kenya where a re-emergence of this sensitivity to CQ was reported after the discontinuation of its uses in 1993 and 1999, respectively [11, 12]. Some studies in Cameroon (Central Africa) and in Senegal (West Africa) [13–15], have reported the same trend for susceptibility to CQ after withdrawal as first-line malaria treatment.

In Côte d’Ivoire, the decreasing clinical efficacy of chloroquine, in addition to a high proportion of resistant isolates, led the Ivorian health authorities to call for withdrawal and discontinuation of the use of chloroquine in 2004 in favor of artemisinin-based combination therapy (ACT) as a first-line treatment for uncomplicated malaria. This study aimed to investigate the prevalence of the K76T mutation a little more than a decade after the official withdrawal of CQ in uncomplicated malaria treatment management in Côte d’Ivoire.

## Methods

### Study site

This was a prospective study that took place in three different health centers, Anonkoua Kouté, Port-Bouët general hospital and Ayamé from February to August 2015. All these sites are located in the southern region of Côte d’Ivoire where the climate is equatorial with annual rainfall exceeding 1700 mm of rain and the temperature varies between 27 and 33 °C. Malaria is seasonal, more frequent during the rainy season from June to September with peaks prevalence rate and incidence in October–November. *Plasmodium falciparum* is the dominant species with more than 90% of malaria parasites identified. The main vectors of malaria in this study area (the southern forest zone of Côte d’Ivoire) are the members of the complexes *Anopheles gambiae* sensu lato (s.l.) and *Anopheles funestus* s.l. [16].

Anonkoua-kouté Health Centre and Ayamé General Hospital were selected based on the high annual incidences of malaria cases records. In addition, these health centers are chosen for several years as the main sites for performing multicenter clinical efficacy tests by the Malaria Unit of Institut Pasteur of Côte d’Ivoire. Port Bouët general hospital was selected for this study not only because of the constantly high annual incidences of malaria cases, but also and especially because of its swampy environment often used for market garden produces.

### Study population and sample collection

All patients clinically suspected to have malaria at Anonkoua Kouté Health Center, Port-Bouët general hospitals and Ayamé during the study period were eligible. However, after informed consent, blood samples were collected from patients who are over 2 years of age with an axillary temperature greater than 37.5 °C and suffering from uncomplicated *P. falciparum* malaria confirmed by microscopic examination.

### Blood sample

In each patient who have been confirmed of having malaria by microscopic examination, approximately 2–5 mL of venous blood was collected in an EDTA tube. Approximately 50 μL of whole blood was dropped on Whatman 3 MM filter paper discs [dried blood spots (DBS)] using a micropipette with filter cones. The papers containing the blood spots were dried for about 60–120 min at room temperature away from dust. Unused blood contained in the EDTA tube was stored in microtubes at − 20 °C for possible subsequent utilization.

### Extraction of *Plasmodium falciparum* genomic DNA

Plasmodial DNA was extracted with methanol from DBS cut into small pieces were immersed in 1 mL of washing buffer (950 μL of 1× PBS plus 50 μL of 10% saponin) and incubated overnight at 4 °C [17]. The wash buffer was removed and 150 μL of methanol were added. After a 20 min incubation, the methanol was gently removed and the samples were dried at room temperature for 2 h before adding 300 μL of sterilized water. The samples were then heated at 99 °C in a thermo-mixer for 30 min to elute the DNA. After removing the confetti debris, the DNA extracts were aliquoted into a 1.5 mL Eppendorf tube and stored at − 20 °C.

### Amplification of the *pfcrt* gene

The pfcrt gene was amplified by nested PCR using a specific pair of primers and a commercial DNA polymerase kit called 5× FIREPol Blend Master Mix with mM MgCl_2_. This kit is a pre-mix (for the reaction mixture) ready to use composed of DNA polymerase (FIREPol^®^ DNA polymerase), buffer (5× Blend Master Mix Buffer), MgCl_2_ (7.5 mM MgCl_2_) and dNTPs (2 mM dNTPs of each). For primary PCR, the primer pairs used for amplification of the *pfcrt* gene were 72_97EF (5′GACCTTAACAGGTGGCTCAC)**/**72_97ER (5′TTTATTGGTAGGTGGAATAG). The primary PCR of this gene was carried out in a reaction volume of 25 μL containing: 0.625 μL of each primer, 3 μL of plasmodial DNA, 5 μL of Taq DNA polymerase and 15.75 μL of milliQ water. The mixture was then put into a PTC-100TM thermocycler (Eppendorf Mastercycler, PTC-100 Peltier Thermal Cycler), programmed as follows: Initial denaturation at 95 °C for 15 min followed by 30 denaturation cycles at 95 °C for 30 s, hybridization at 58 °C for 2 min and extension at 72 °C for 2 min. Finally, a terminal extension at 72 °C for 10 min.

The second PCR was carried out on the amplification products of the primary PCR in a reaction volume of 50 μL containing: 1.25 μL of each primer, 5 μL of amplification product (amplicon) of the first PCR, of 5 μL of Taq DNA polymerase and 37.5 μL of milliQ water. The primer pairs used for the secondary PCR were SecIF (5′ GGTAAATGTGCTCATGTGTTTAAACTTATT)/SecIR (5′ TTACTTTTGAATTTCCCTTTTTATTTCCA). The secondary PCR was performed with the same thermocycler used for the primary PCR with the following program: Initial denaturation at 95 °C for 15 min followed by 30 denaturation cycles at 95 °C for 30 s, hybridization at 60 °C for one minute and extension at 72 °C for 1 min. Finally, a terminal extension at 72 °C for 10 min.

### Detection and analysis of PCR products

The amplification products were migrated on a 1.5% agarose gel containing ethidium bromide (EtBr). After migration, the gel was recovered and then observed under a UV lamp using the UV transilluminator (Gel DocTM EZ Imager). The presence or absence of bands made it possible to judge the effectiveness of the PCR.

### Sequencing amplification

The amplified DNA fragments (*pfcrt* gene) of *P. falciparum* were sequenced according to the Sanger method by the company Eurofins MWG operon (Cochin sequencing platform). Samples were dropped to the platform in a microplate (Greiner Bio-one-652270B) along with a deposit slip that was sent to the platform’s email address. A reaction medium was prepared for the PCR-nested sense primer (sequencing primer) from the amplification products. In each well of the microplate, a volume of 13 μL of amplification product was added to 2 μL of sequencing primer at 10 μM. Wells containing the sequencing reaction medium were sealed with cap strips (4titude-044737) before covering the entire surface of the microplate with an adhesive film (AmpliSeal, Greiner Bio-one-676040). This microplate containing the samples was sent to the platform for sequencing.

After the sequencing reaction, the received DNA sequences were recovered as fasta. In this study, it is the sequences corresponding to the *pfcrt* gene of the isolates collected. The use of the software BioEdit made it possible to analyse the sequences to search for possible mutations. Indeed, the loci of interest, namely the codons at position 74, 75, 76 of the *PfCRT* polypeptide or the nucleotides at position 222, 225, 228 of the *pfcrt* gene were identified and analysed after a parallel alignment of two or more DNA sequences, including the reference sequence *pfcrt* gene, by maximizing the number of identical nucleotides or residues, while minimizing the number of mismatches and voids.

### Statistical analysis of data

Data were collected on a standard questionnaire tested and validated. They were then entered and analysed on the statistical software R; version 3.2.2 [18]. The χ^2^ comparison test of three averages was used to compare the prevalence of the molecular marker of CQ resistance (*pfcrt* K76T). The χ^2^ test was used to determine whether the molecular marker prevalence can be considered to be all equal (null hypothesis H0) or if two or more prevalence are different (alternative hypothesis Ha). A difference and/or statistical association was considered significant if p of the test χ^2^ < 0.05.

## Results

**Fig. 1:**
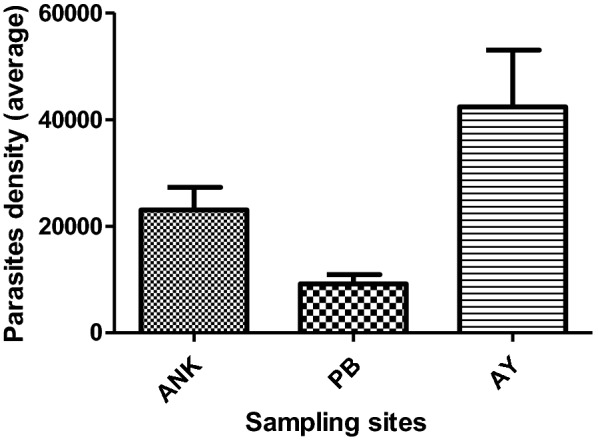
Mean parasite density versus sampling site. *ANK* Anonkoua-Kouté, *PB* Port-Bouët, *AY* Ayamé

A total of 64 persons infected with *P. falciparum* were selected for this study, including 41 (64%) women and 23 (36%) men. The average age of the patients was 17 years (age ranging from 2 to 62 years) (Table 1). In addition, the parasite densities varied from 1200 to 200,000 parasites/μL with average parasite densities of 22,900; 9193 and 42,327 parasites/μL at Anonkoua Kouté, Port-Bouët and Ayamé, respectively (Table 1 and Fig. 1). A significant difference (p = 0.002) was observed between the parasite densities at Port-Bouët and Ayamé. The mean parasite density in all three sites was 24,806 parasites/μL.

**Table 1:** Samples used for molecular analysis of chloroquine chemoresistance

Sites	Period of collection in 2015	Age group (years)	DBS collected	Average Parasite density (µL/mm^3^)
Anonkoua-kouté	February–March	2–53	28	22,900
Port-Bouët	April–May–July	2–62	16	9193
Ayamé	June–July–August	2–55	20	42,327
Total			64	24,806

### Prevalence of the individual alleles of the *pfcrt* gene and molecular analysis of the corresponding genotypes

Across all the three study sites, the results indicated that the prevalence of wild-type isolates Met-74 (73%), Asn-75 (75%), Lys-76 (62%) is higher than those isolates carrying mutations of the *pfcrt* gene Ile-74 (7%), Glu-75 (7%) and Thr-76 (15%) (Table 2). Molecular analysis of the genotypes corresponding to the *pfcrt* gene shows that the MNK genotype (wild type) was predominant with a prevalence of 62% (Table 3). In contrast, single genotypes mutant, double mutant and triple mutant were observed with respective prevalence of 12%, 6% and 18%.

**Table 2:** Prevalence of the individual alleles of the *pfcrt* gene in the study sites

Codons	Alleles	Sample size (N = 64)	
		**n = 59**	**%**
Crt_74	Wild		
	Met-74	47	73
	*Ile-74*	5	7
	Mutants		
	Lys-74	1	1
	Leu-74	5	7
	Trp-74	1	1
Crt_75		**n = 64**	**%**
	Wild		
	Asn-75	48	75
	Glu-75	5	7
	Mutants		
	Lys-75	6	9
	Tyr-75	5	7
Crt_76		**n = 64**	**%**
	Wild		
	*Lys-76*	40	*62*
	*Thr-76*	10	*15*
	Mutants		
	Gly-76	1	1
	Ile-76	1	1
	Gln-76	16	25

“N” represents the total number of isolates sequenced at the three sites. “n” represents the number of isolates sequenced successfully by codon

**Table 3:** Prevalence of genotypes corresponding to *pfcrt*

*pfcrt* key codons	Genotypes			Sample size (N = 64)
	M74I	N75E	K76T	n (%)
Wild type	M	N	K	*40 (62)*
Single mutations				*8 (12)*
	M	N	*T*	6 (9)
	Others			2 (3)
Double mutations	M	*Y*	*Q*	*4 (6)*
Triple mutations				*12 (18)*
	*L*	*K*	*Q*	6 (9)
	*I*	*E*	*T*	4 (6)
	*K*	*E*	*G*	1 (1)
	*I*	*Y*	*I*	1 (1)

A capital letter in the “genotype” column represents the one-letter code of amino acids. The amino acids resulting from the mutation of *pfcrt* are underlined and in italic. The determined prevalences correspond to the number of observations on the number of successes per gene

Among the triple mutant genotypes, there was a predominance of isolates carrying IET and LKQ with respective prevalence of 6% and 9%. The analysis also found single mutant MNT (9%) and double mutant MYQ (6%) genotypes (Table 3).

### Prevalence of wild-type Lys-76 and Thr-76 mutants of the *pfcrt* gene at Anonkoua-Kouté, Port-Bouët and Ayamé

No Thr-76 mutation was observed in Anonkoua-Kouté while this mutant allele was found in 31% and 25% of the sequences of the isolates from Port-Bouët and Ayamé, respectively, as indicated in Table 4. The highest prevalence of the Thr-76 allele were found in Port-Bouët (31%) and Ayamé (25%) (Table 4). Moreover, in all the study sites no significant difference (P = 0.955) was observed in the prevalence of the wild-type Lys-76 allele, Anonkoua-Kouté (60%), Port-Bouët (62%) and Ayamé (65%).

**Table 4:** Prevalence of the wild-type Lys-76 and the mutant Thr-76 of the *pfcrt* gene in Anonkoua-Kouté, Port-Bouët and Ayamé

Codon	Alleles	Anonkoua-Kouté: N = 28	Port-Bouët: N = 16	Ayamé: N = 20	p-value (χ^2^ test)
		n (%)	n (%)	n (%)	
Lys-76-Thr	Lys-76	17 (60)	10 (62)	13 (65)	0.955
	*Thr-76*	*0 (0)*	*5 (31)*	*5 (25)*	*–*

The mutated amino acids are italic. “N” represents the total number of isolates sequenced successfully per study site. “n” represents the number of isolates sequenced successfully at the codon Crt_76

The list of other mutants is in Table 2

The χ^2^ test could not be performed for the mutants because of the value less than 5 in a cell

## Discussion

Previous studies carried out in West Africa and particularly in Côte d’Ivoire showed a strong correlation between the Thr-76 mutation of the *pfcrt* gene and the therapeutic failures on one hand, and between the Thr-76 mutation of the *pfcrt* gene and in vitro chemoresistance of *P. falciparum* isolates to chloroquine [19–22] on the other hand.

Results indicate that in all three study sites, the mutant allele Thr-76 (15%) was associated with mutant Ile-74 (7%) and Glu-75 (7%) in isolates at very low proportion compared to wild-type alleles Lys-76 (62%), Met-74 (73%) and Asn-75 (75%). In addition, the wild allele Lys-76 was observed in Anonkoua-Kouté, Port-Bouët and Ayamé 60%, 62% and 65%, respectively.

These results contrasted those obtained (for the Thr-76 mutation) in 2006 (65%) in Yopougon, Abidjan [8], in 2005 (100% and 95%) in Bonoua and Samo respectively [10] and in 2010 (62%) in Adzopé [9]. However, the data are in accordance with Gharbi et al. [23] who, after modelling chloroquine resistance in Côte d’Ivoire from the Thr-76 allele with samples from travelers returning to Côte d’Ivoire reported a decrease in prevalence from 63 to 37% of Thr-76 allele.

Normally, the prevalence of the Thr-76 mutation should be reduced because parasites that carry the Lys-76 wild-type allele have a survival advantage in the absence of drug pressure [24]. Indeed, when drug pressure is low, drug resistance is accompanied by a reduction in the genetic performance of resistant parasites compared to susceptible parasites [25, 26]. Thus, when the drug pressure decreases, the proportion of sensitive parasites increases and that of the resistant parasites decreases [11].

The low prevalence observed for Thr-76 isolates may be due to a number of factors, the main one being the effective withdrawal of CQ in Côte d’Ivoire. Indeed, CQ has always been prescribed and/or delivered in Côte d’Ivoire for the treatment of uncomplicated malaria [27] until 2007, when it was withdrawn in favor of amodiaquine (AQ) [28]. However, the substitution of amodiaquine would have delayed the decline since AQ also selects for the Thr-76 allele of *pfcrt* [29] in contrary to the countries where the decline has been most precipitous thanks to an intensive deployment of lumefantrine as part of Coartem. The data generated are in line with these observations, since artemether-lumefantrine (AL) was officially used as first-line treatment in Côte d’Ivoire by year 2013. It was, therefore, necessary to wait a few years to observe a possible significant decrease in chloroquine-resistant *P. falciparum* isolates. Thus, the public authorities have had to intensify awareness campaigns to inform the population and the medical staff, to take action to fight against the illegal sale of anti-malarial drug on parallel markets (street drugs). All these actions eventually made effective the removal of the CQ and that would be the basis of the increase in the prevalence of chloroquine-sensitive parasites. In addition, government control over pharmaceutical distribution channels and drug supply chains in the public and private sectors has reduced the use of non-recommended drugs such as CQ [30, 31]. For example, in Malawi, the successful implementation of national information campaigns and the effective control of drug delivery patterns has led, 10 years later, to the re-emergence of sensitivity to CQ [32]. The context in Côte d’Ivoire is also different from what was reported in French Guyana where, despite the fixation of the IET genotype, the return to sensitivity was observed thanks to the acquisition of the C350R mutation in parasites carrying the Lys-76 allele [33]. No mutation of the Thr-76 allele was observed in Anonkoua Kouté while this mutant allele was carried by 31% and 25% of isolates from Port-Bouët and Ayamé, respectively.

These prevalence of the Thr-76 mutation in Port-Bouët and Ayamé could be related to the effects of migratory movements of the populations towards these areas. Indeed, these two communities are characterized by a strong agroeconomic activity (livestock, farming, and fishing) with many rivers, large farms that attract many indigenes and non-indigenes from the sub-region. These populations mainly occupy the villages (Ayamé) and the many precarious neighbourhoods (Port-Bouët) where most households often do not have access to national information thus depriving them of awareness campaigns for the withdrawal and abandon of the CQ. Thus, these households opt for anti-malarial treatments without consultation (self-medication), thus maintaining drug pressure [34]. The populations migrating from neighbouring West African countries to these localities may also be potential carriers of resistant parasites, which could explain the prevalence of the Thr-76 mutation observed in these areas.

Therefore, the withdrawal of chloroquine and the introduction of ACT seem to promote the re-emergence of CQ-sensitive isolates. It would be desirable to carry out another study that could be extended to several localities with a larger number of samples to confirm this decrease of CQ-resistant parasites in Côte d’Ivoire. Thus, if the proportion of chloroquine-resistant parasites decreases at the national level to an undetectable level of *pfcrt* mutants, a reintroduction of chloroquine in combination with other anti-malarial drug for malaria treatment and prophylaxis may be considered, as Malawi has done [11].

## Conclusion

The present study showed that the CQ resistance has decreased in Côte d’Ivoire in Anonkoua-Kouté, Port-Bouët and Ayamé communities since its withdrawal in 2004. This decrease in CQ resistance seems to be related to the efficiency and the success of the policy of abandoning the use of CQ in Côte d’Ivoire. Therefore, the withdrawal of chloroquine (CQ) and the introduction of ACT for the treatment of uncomplicated malaria in Côte d’Ivoire appear to favour the re-emergence of isolates sensitive to CQ. However, even if the proportion of chloroquine-sensitive parasites seems to increase in Anonkoua-Kouté, Port-Bouët and Ayamé, a reintroduction of chloroquine in malaria treatment cannot be recommended currently in Côte d’Ivoire.

## Additional files

**Additional file 1:** Data on sampling sites and on subjects included in the study.

## Abbreviations

ACT: artemisinin-based combination therapy
AQ: amodiaquine
CQ: chloroquine
CQR: chloroquine resistance
DNA: deoxyribonucleic acid
EDTA: ethylene diamine tetraacetic
PBS: phosphate buffered saline
PCR: polymerase chain reaction
NCER: National Committee on Ethics and Research
WHO: World Health Organization
